# “Utility of Cerebrospinal Fluid Unstimulated Interferon-Gamma (IRISA-TB) as a Same-Day Test for Tuberculous Meningitis in a Tuberculosis-Endemic, Resource-Poor Setting”

**DOI:** 10.1093/ofid/ofaf269

**Published:** 2025-05-05

**Authors:** Tessa Adzemovic, Fiona V Cresswell, Wubbo de Boer, Mahomed-Yunus S Moosa, Nathan C Bahr

**Affiliations:** Division of Hospital Medicine, Department of Medicine, Brigham and Women's Hospital, Boston, Massachusetts, USA; Infectious Diseases Institute, Makerere University, Kampala, Uganda; Infectious Diseases Institute, Makerere University, Kampala, Uganda; HIV Interventions, Medical Research Council–Uganda Virus Research Institute, and London School of Hygiene & Tropical Medicine Uganda Research Unit, Entebbe, Uganda; Global Health and Infection, Brighton and Sussex Medical School, Brighton, UK; Department of Infectious Diseases, University College London Hospitals NHS Foundation Trust, London, UK; Department of Infectious Disease, Nelson R. Mandela School of Medicine, University of KwaZulu-Natal, Durban, South Africa; Division of Infectious Diseases and International Medicine, Department of Medicine, University of Minnesota, Minneapolis, Minnesota, USA

To the  Editor—We read with great interest the recently published article by Randall et al titled “Utility of Cerebrospinal Fluid Unstimulated Interferon-Gamma (IRISA-TB) as a Same-Day Test for Tuberculous Meningitis in a Tuberculosis-Endemic, Resource-Poor Setting.”

We applaud the investigators for their efforts to evaluate same-day host-based diagnostics for tuberculous meningitis (TBM) and agree that improved TBM diagnostic testing remains a priority and would likely improve mortality in this vulnerable population.

However, we would like to highlight that in diagnostic accuracy studies that lack a perfect gold standard reference test, as is the case with TBM, consistency among studies is important. For this reason, research consensus case definitions (ie, the Marais uniform criteria) were developed in 2010 for TBM [1]. These criteria were developed to standardize results, allow comparison between studies, and enable the pooling of data for systematic reviews and meta-analyses. Although the authors attest that one of the strengths of the study was that patients were categorized by strict definitions, their categories do not align with the Marais uniform criteria. For instance, in the authors’ definitions, probable or definite TBM “must have responded to therapy,” which does not reflect the natural history of TBM, as many cases do not respond to therapy and result in death or disability. Response to therapy is not part of the published consensus case definitions. As presented, it is not possible to accurately interpret the accuracy of diagnostic tests due to likely misclassification.

Additionally, prior epidemiologic studies in similar African HIV/TB-endemic settings have shown that approximately 10% to 15% of persons presenting with meningitis will have definite/probable TBM [2, 3]. In stage 2 of this study, only 4% (29/686) had definite/probable TBM per the authors’ nonstandard definitions. The small proportion and the use of nonstandard definitions further raise concern for misclassification.

Last, we disagree with the authors' contention that the Xpert Ultra trace results were “false positives.” The authors used Sanger sequencing of the cartridge-generated amplicon. While trace TB DNA may persist in the respiratory microbiome after prior treatment and may cause false-positive results, this is not the case for cerebrospinal fluid (CSF), where the presence of TB DNA indicates the presence of disease in symptomatic patients [4, 5]. Additionally, no method, including Sanger sequencing, can detect every case of TBM [4]. In a person suspected to have TBM, a negative Sanger sequencing result from a sample positive by Xpert or Xpert Ultra is indicative of a falsely negative Sanger sequencing result rather than a falsely positive detection of TB DNA. Interpretation of trace-positive Xpert Ultra results must take into account the entire clinical picture. Given the dire consequences of not treating TB meningitis, symptomatic persons with trace-positive Xpert Ultra CSF results should nearly always be treated.

In conclusion, while we concur that the evaluation of IRISA-TB on CSF is very relevant, the investigators' categorization of participants should be revisited to allow appropriate comparison with current available tests.

## Notes

***Author contributions.*** T. A., F. V. C., and N. C. B. participated in the conceptualization of the manuscript. T. A. completed writing of the original draft. F. V. C., W. D. B., M.-Y. M., and N. C. B. completed writing–review and editing.

***Financial support*.** This work was supported by the National Institute of Allergy and Infectious Diseases (T32AI055433, R01AI170158); the National Institute of Neurological Disorders and Stroke (K23110470); and Wellcome Trust (300088/Z/88/Z).
